# Stemness and Cell Cycle Regulators and Their Modulation by Retinoic Acid in Ewing Sarcoma

**DOI:** 10.3390/cimb46050246

**Published:** 2024-04-26

**Authors:** Maria Eduarda Battistella, Natália Hogetop Freire, Bruno Toson, Matheus Dalmolin, Marcelo A. C. Fernandes, Isadora D. Tassinari, Mariane Jaeger, André T. Brunetto, Algemir L. Brunetto, Lauro Gregianin, Caroline Brunetto de Farias, Rafael Roesler

**Affiliations:** 1 Cancer and Neurobiology Laboratory, Experimental Research Center, Clinical Hospital (CPE-HCPA), Federal University of Rio Grande do Sul, Porto Alegre 90035-003, Brazil; 2 Children’s Cancer Institute, Porto Alegre 90620-110, Brazil; 3 National Science and Technology Institute for Children’s Cancer Biology and Pediatric Oncology—INCT BioOncoPed, Porto Alegre 90035-003, Brazil; 4 InovAI Lab, nPITI/IMD, Federal University of Rio Grande do Norte, Natal 59078-970, Brazil; 5 Bioinformatics Multidisciplinary Environment (BioME), Federal University of Rio Grande do Norte, Natal 59078-970, Brazil; 6 Department of Computer Engineering and Automation, Federal University of Rio Grande do Norte, Natal 59078-970, Brazil; 7 Laboratory of Neurobiology and Metabolism (NeuroMet), Department of Physiology, Institute for Basic Health Sciences, Federal University of Rio Grande do Sul, Porto Alegre 90035-003, Brazil; 8 Graduate Program in Physiology, Institute for Basic Health Sciences, Federal University of Rio Grande do Sul, Porto Alegre 90035-003, Brazil; 9 Department of Pediatrics, School of Medicine, Federal University of Rio Grande do Sul, Porto Alegre 90035-003, Brazil; 10 Pediatric Oncology Service, Clinical Hospital, Federal University of Rio Grande do Sul, Porto Alegre 90035-003, Brazil; 11 Department of Pharmacology, Institute for Basic Health Sciences, Federal University of Rio Grande do Sul, Porto Alegre 90035-003, Brazil

**Keywords:** retinoic acid, SK-ES-1 cell, stemness, differentiation, Ewing sarcoma

## Abstract

Retinoic acid (RA) regulates stemness and differentiation in human embryonic stem cells (ESCs). Ewing sarcoma (ES) is a pediatric tumor that may arise from the abnormal development of ESCs. Here we show that RA impairs the viability of SK-ES-1 ES cells and affects the cell cycle. Cells treated with RA showed increased levels of p21 and its encoding gene, *CDKN1A*. RA reduced mRNA and protein levels of SRY-box transcription factor 2 (SOX2) as well as mRNA levels of beta III Tubulin (*TUBB3*), whereas the levels of *CD99* increased. Exposure to RA reduced the capability of SK-ES-1 to form tumorspheres with high expression of SOX2 and Nestin. Gene expression of *CD99* and *CDKN1A* was reduced in ES tumors compared to non-tumoral tissue, whereas transcript levels of *SOX2* were significantly higher in tumors. For *NES* and *TUBB3*, differences between tumors and control tissue did not reach statistical significance. Low expression of *CD99* and *NES*, and high expression of *SOX2*, were significantly associated with a poorer patient prognosis indicated by shorter overall survival (OS). Our results indicate that RA may display rather complex modulatory effects on multiple target genes associated with the maintenance of stem cell’s features versus their differentiation, cell cycle regulation, and patient prognosis in ES.

## 1. Introduction

Retinoic acid (RA) is a vitamin A derivative that displays actions as an endogenous signaling molecule by modulating cells’ development and function. RA acts through three RA receptors (RARs), namely RAR-α, RAR-β, and RAR-γ, which constitute a family of transcription factors that regulate gene expression and form heterodimers with retinoid X receptors (RXRs), RXR-α, RXR-β, and RXR-γ to bind RA response elements (RAREs) in target gene promoters [1,2]. RA is critically involved in embryonic stem cell (ESC) development, at least partially by modulating epigenetic changes through functional interactions with histone deacetylases (HDACs) [3,4].

RA has been used as a biochemical tool to promote the differentiation of neuroblastoma (NB) cells, particularly the SH-SY5Y cell line [5,6,7,8]. Early research on RA in NB included pioneer work by Thiele et al. who reported that treating human NB cell lines with RA promotes neurite outgrowth, cell cycle changes, and a reduction in the expression of N-*myc*, overall driving the cells towards a more differentiated state and a neuronal-like phenotype [9,10]. Studies such as this have ultimately led to the clinical testing of RA-related retinoids, particularly the isomer 13-cis-retinoic acid (isotretinoin), as pro-differentiating therapies for the treatment of pediatric patients with NB [11,12,13,14]. In addition, RA-differentiated SH-SY5Y cells have been established as a useful in vitro model of human neurons which is suitable to be used as an experimental platform for the study of neurodegeneration associated with diseases such as Alzheimer’s disease and Parkinson’s disease [15,16,17].

Like NB, Ewing sarcoma (ES) is a type of solid pediatric cancer that may arise from disruptions in epigenetic regulation or cell signaling during ESC [18]. Although still debated, it is hypothesized that ES cells might have a neural origin, possibly from fetal neural crest cells [19,20], and carry the potential for neuronal differentiation [21,22]. Despite its well-known differentiation-promoting effects in NB, RA can stimulate either differentiation or stemness depending on the cell type. Moreover, RA can also influence other aspects of cancer cell function, including proliferation and survival [23].

Considering the impact of RA treatment in NB-related studies, we believe further assessment in other pediatric cancers could shed light on aspects of cancer cell function. As it remains poorly understood how RA can affect ES cells, in the present study we looked into effects of RA treatment on SK-ES1 human ES cells and the role of genes modulated by RA in ES.

## 2. Materials and Methods

### 2.1. Cell Culture

SK-ES-1 cells were obtained from the American Type Culture Collection (ATCC; Rockville, MD, USA) and checked for authenticity and contamination. Cells were grown in RPMI (Gibco, Grand Island, NE, USA) supplemented with 10% fetal bovine serum (FBS, Gibco), 1% penicillin and streptomycin (Gibco), and 0.01% amphotericin B (Gibco). Exponentially growing cells were detached with 0.25% trypsin solution. Cells were maintained at 37 °C with a humidified atmosphere and 5% CO_2_.

### 2.2. Cell Viability

Cells were seeded at 3000 cells/well in 96-well plates. After 24 h, cells were treated with 5 or 10 µM of RA (Sigma-Aldrich, St. Louis, MO, USA) and retreated after 72 h given that RA degrades easily and thus its effect is reduced over time. After seven days of RA exposure, cells were detached and counted in a Neubauer chamber with trypan blue (10 µL of dye for each 10 µL of cell suspension) for viability measurement.

### 2.3. Cell Survival

To evaluate the effects of RA on cell survival capability, cells were treated for seven days with 5 or 10 µM of RA. After this period, 1000 cells/well were plated in 6-well plates in a treatment-free medium. After a period of seven days, cells were fixed in 70% ethanol and counterstained with 0.1% crystal violet. Stained colonies were counted and measured using ImageJ (version 1.54d, National Institutes of Health, Bethesda, MD, USA).

### 2.4. Cell Cycle

To assess the cell cycle, SK-ES-1 cells were treated with RA and detached, centrifuged, and washed with PBS. The cells were then re-suspended in 50 μg/mL propidium iodide (PI) (Sigma-Aldrich) in 0.1% Triton X-100 and 0.1% sodium citrate solution. Cells were stained in PI cell cycle solution for at least 15 min followed by assessment on an Attune Acoustic focusing cytometer by Applied Biosystems (Thermo Fisher Scientific, Waltham, MA, USA). In each sample, 20,000 cells were analyzed. Analysis was performed using AttuneTm Next Acoustic Focusing (Life Technologies, Carlsbad, CA, USA).

### 2.5. Reverse Transcriptase Polymerase Chain Reaction (RT-qPCR)

RNA was extracted using ReliaPrep™ RNA Miniprep Systems (Promega, Madison, WI, USA) and quantified by spectrophotometer NanoDrop in 260 nm wavelength. For cDNA synthesis, we used GoScript™ Reverse Transcription System (Promega). The transcriptional levels of target genes cyclin dependent kinase inhibitor 1A (*CDKN1A*), SRY-box transcription factor 2 (*SOX2*), Nestin (*NES*), beta III Tubulin (*TUBB3*), and *CD99* were quantified using PowerUp™SYBR™ Green Master Mix (Thermo Fisher Scientific) in the QuantStudio 5 Real-Time PCR System (Thermo Fisher Scientific). Expression of β-actin was used as an internal control. Primer sequences are described in Table 1.

### 2.6. Western Blot

Cells treated with RA and controls were lysed with 1× lysis buffer (CellLysis Buffer, Cell Signaling Technology, Danvers, MA, USA), and protein was quantified using the Bradford protein assay (Thermo Fisher Scientific). For blotting, 20 μg of protein were separated by SDS-PAGE and transferred to a PVDF membrane. After 1 h under blocking solution (5% Milk in TTBS), the membrane was incubated overnight at 4 °C with primary antibodies against SOX2 (ab97959, 1:2000 dilution; Abcam, Cambridge, UK), p21 (sc-6246, 1:500 dilution; Santa Cruz Biotechnology, Dallas, TX, USA), and β-actin (ACTB) (sc-47778, 1:2000 dilution; Santa Cruz Biotechnology) as protein control. Incubation of primary antibodies was followed by incubation with secondary antibodies (1:10,000 dilution; anti-rabbit IgG 7074, Cell Signaling; or anti-mouse IgG a4416, Sigma-Aldrich) for 1 h. Chemiluminescence was detected using ECL Western Blotting Substrate (Thermo Fisher Scientific) and analyzed using iBright (Thermo Fisher Scientific). The immunodetection signal was analyzed using ImageJ.

### 2.7. Tumorsphere Formation

A tumorsphere expansion assay was performed as previously described [24]. Briefly, cells were plated at 1000 cells/well in 24-well plates using DMEM/F12 (1:1) sphere induction medium containing 2% of B27 (Gibco), 20 ng/mL recombinant human bFGF (Sigma-Aldrich), 20 ng/mL recombinant human EGF (Sigma-Aldrich), heparin 10 IE/mL (5 mg/mL) (Roche, Mannheim, Germany), and antibiotics. After three days of sphere formation, RA was added at 10 μM concentration. Sphere photomicrographs were captured at day seven under an inverted phase microscope (Leica Microsystems, Mannheim, Germany) at ×5 magnification. Spheres were measured using ImageJ. The spheres’ RNAs were also collected for RT-qPCR analysis.

### 2.8. Gene Expression

Expression data for *CD99*, *CDKN1A*, *NES*, *SOX2*, and *TUBB3* were acquired from the Gene Expression Omnibus (GEO) [PMC4944384]. We used the dataset GSE17679, GPL570 Affymetrix Human Genome U133 Plus 2.0 Array, which contains gene expression data from 44 ES tumor patients and 18 normal muscle samples which are used as controls [25]. Raw microarray data normalization was performed using the Robust Multichip Average (RMA) method through the Affy Bioconductor/R package [PMID: 14960456]. Clinical information was obtained through the ‘geoquery’ package. GPL570 annotations were downloaded from the database: https://www.ncbi.nlm.nih.gov/geo/query/acc.cgi?acc=GPL570 (accessed on 10 March 2024).

### 2.9. Statistical Analysis

Data from cellular and molecular experiments are shown as mean ± standard deviation (SD). Statistical analysis was performed by one-way analysis of variance (ANOVA) followed by Bonferroni’s post hoc tests for multiple comparisons. To analyze two conditions, unpaired test T was used. Experiments were replicated at least three times; *p* values under 0.05 were considered significant. The GraphPad Prism 8 software 10.1.2 (GraphPad Software, San Diego, CA, USA) was used for the analyses. Differences in expression between ES and normal tissue (muscle) for each of five genes (*CD99*, *CDKN1A*, *NES*, *SOX2*, and *TUBB3*), were analyzed with a Mann–Whitney test. Statistically significant differences were verified using the Holm-adjusted *p*-value test, and analyses were performed using the ‘ggstatsplot’ package. To investigate the relationship between gene expression levels and overall survival (OS) of ES patients, the “Survminer” package with ‘minprop = 0.2’ was used to classify patients into high and low gene expression groups, and survival analysis was conducted using the “Survival” package.

## 3. Results

### 3.1. RA Impairs SK-ES-1 Cell Viability but Not Colony-Forming Capacity

**Figure 1:**
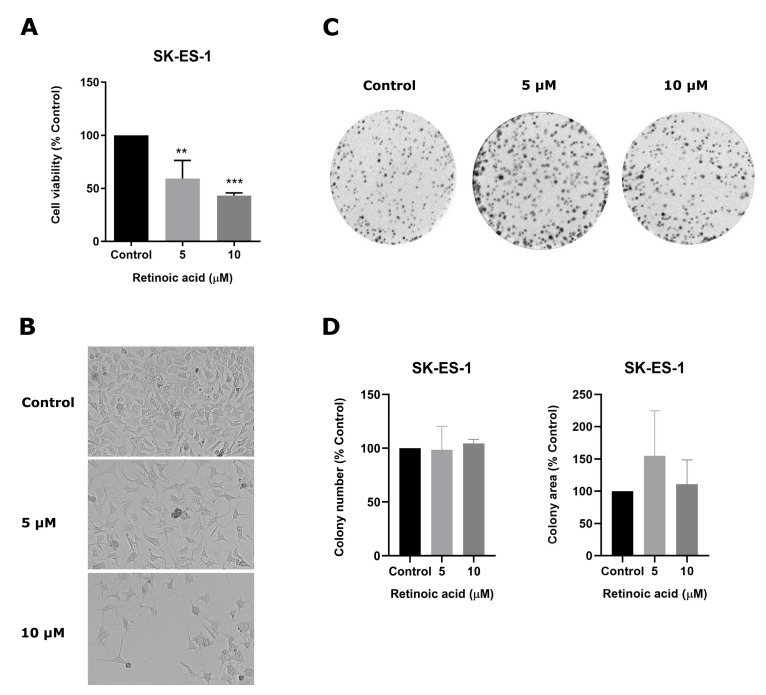
RA reduces SK-ES-1 cell viability without changing cell survival. (**A**) Cell viability of RA-treated and control SK-ES-1 cells was verified using trypan blue exclusion assays. (**B**) Representative images of cells after RA exposure, taken using a an inverted microscope with 10× magnification (**C**) Representative images of crystal violet staining of RA-treated and control cell colonies after seven days of growth in a RA-free medium. (**D**) Colony formation measured by colony number and size. Results represent the mean ± SD of three independent experiments; ** *p* < 0.01; *** *p* < 0.001 compared to controls.

To evaluate the effects of RA on cell viability, we exposed SK-ES-1 cells to RA at 5 or 10 µM for seven days. Both RA concentrations significantly reduced the number of live cells: RA at 5 µM induced a 40.6% reduction and RA at 10 µM induced a 56.7% reduction compared to controls (*p* < 0.01 and *p* < 0.001; Figure 1A,B). To assess whether RA has a long-term impact on cell survival and proliferation, we next assessed their ability to form colonies by growing previously treated cells in a drug-free medium for seven days. The results reveal that the number and area of colonies were not significantly changed by previous RA treatment (Figure 1C,D).

### 3.2. RA Modulates Cell Cycle Progression in SK-ES-1 Cells

**Figure 2:**
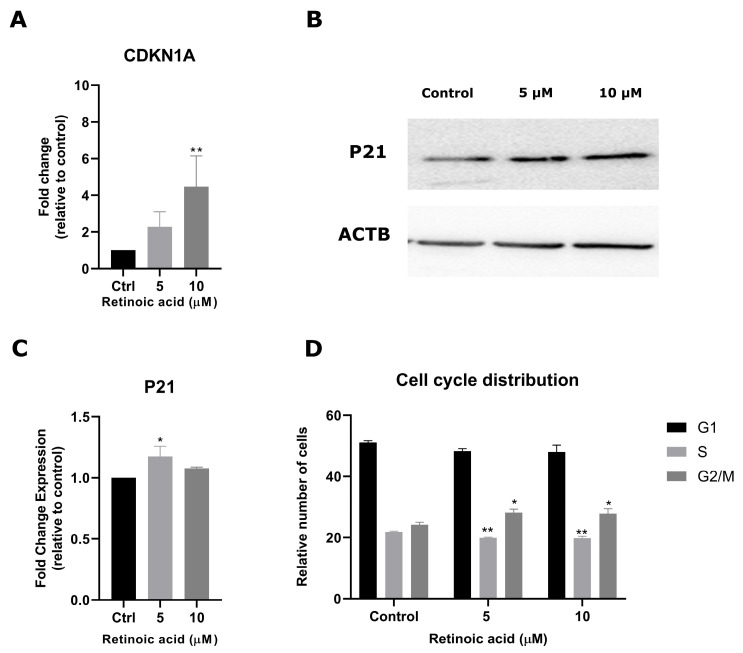
RA regulates SK-ES-1 cell cycle progression. (**A**) Relative mRNA levels of *CDKN1A* in RA-treated and control cells were verified using RT-qPCR. (**B**) Western blot analysis of p21 after RA exposure. (**C**) Relative Densitometric Unit (RDU) analysis normalized by *ACTB* and corrected by control. (**D**) Cell cycle distribution of SK-ES 1 after seven days of RA exposure. Results represent the mean ± SD of three independent experiments; * *p* < 0.5; ** *p* < 0.01 compared to controls.

To further investigate RA’s effects on cell function, we next evaluated cycle regulation by analyzing *CDKN1A* (the gene that encodes p21) levels after RA treatment. Exposure to 10 µM of RA resulted in a 3.4-fold increase in *CDKN1A* levels (*p* < 0.01) (Figure 2A). Protein levels of p21 showed an increase of 17% after exposure to 5 µM of RA (*p* < 0.05) (Figure 2B,C). RA exposure induced statistically significant but modest effects on the cell cycle, namely a small reduction in the number of cells in the S phase (*p* < 0.01) and increase ini G2/M arrest at both RA concentrations (*p* < 0.05 compared to controls) (Figure 2D).

### 3.3. RA Influences Expression of Stemness Regulators in SK-ES-1 Cells

**Figure 3:**
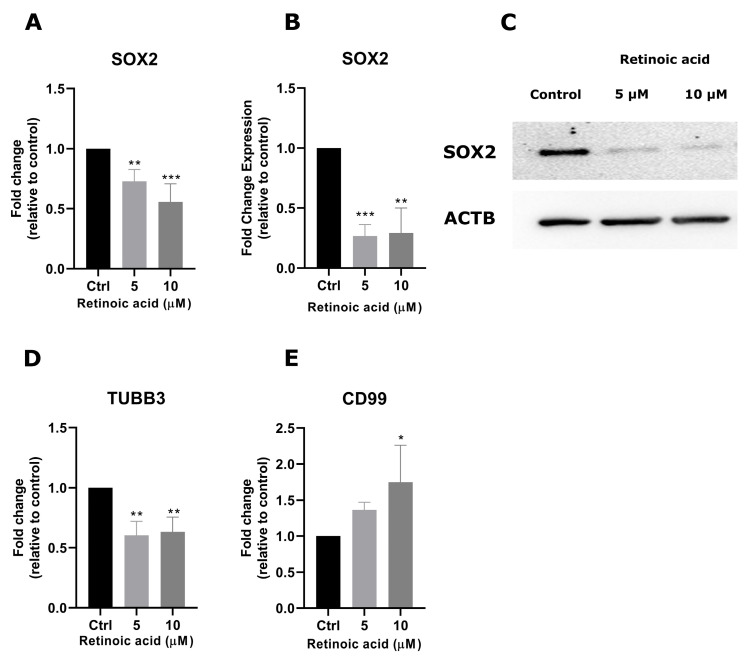
RA modulates stemness regulators in SK-ES-1 cells. (**A**) Relative mRNA levels of *SOX2* in RA-treated and control cells were assessed with RT-qPCR. (**B**) Western blot analysis of SOX2 protein after RA exposure. (**C**) Relative Densitometric Unit (RDU) analysis normalized by *ACTB* and corrected by controls. Relative mRNA levels of (**D**) *TUBB3* and (**E**) *CD99* were determined in control and RA-treated SK-ES-1 cells by RT-qPCR. Relative mRNA levels were analyzed by the delta CT method. Results represent the mean ± SD of three independent experiments; * *p* < 0.05; ** *p* < 0.01; *** *p* < 0.001 compared to controls (Ctrl).

To investigate the molecular pathways associated with the antiproliferative effects of RA, we evaluated the transcriptional levels of stemness regulatory RA target genes *SOX2*, *CD99*, and *TUBB3*. RA exposure reduced *SOX2* transcriptional levels at 5 and 10 µM (0.27-fold, *p* < 0.01; 0.44-fold, and *p* < 0.001, respectively; Figure 3A). Accordingly, the protein levels of SOX2 were also decreased after RA exposure at 5 and 10 µM (73.2%, *p* < 0.001; 70,7% *p* < 0.01; Figure 3B,C). In addition, RA exposure decreased the transcriptional levels of *TUBB3* at 5 and 10 µM (0.39-fold; 0.36-fold, respectively; *p* < 0.01) and caused an increase in *CD99* levels at the 10 µM concentration (0.75-fold, *p* < 0.05) (Figure 3D,E).

### 3.4. RA Reduces Tumorsphere-Forming Capacity in SK-ES-1 Cells

**Figure 4:**
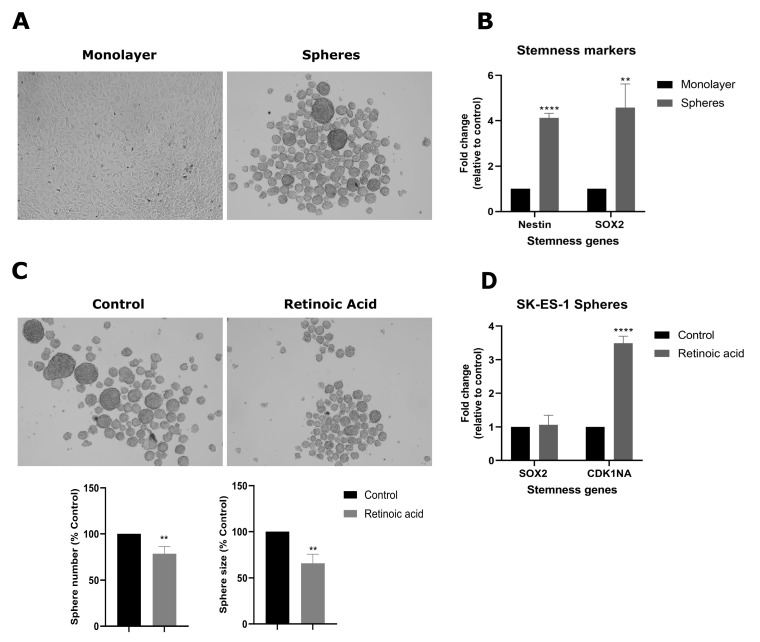
RA inhibits SK-ES-1 cell tumorsphere formation. SK-ES-1 cells were cultured in a specific medium for tumor stem cell expansion over 7 days for enrichment of spheres. (**A**) Representative images of SK-ES-1 tumorspheres and cells in monolayers, taken using a an inverted microscope with 10× magnification (**B**) Relative mRNA levels of stemness markers SOX2 and Nestin were determined in monolayers and tumorsphere cultures by RT-qPCR using specific primers. (**C**) Representative photomicrographs of tumorspheres after 7 days of induction in the absence (controls) or presence of RA at 10 µM, taken using a an inverted microscope with 10× magnification. RA was added on the third day of sphere induction. Relative number and size of RA-treated and control tumorspheres. (**D**) Relative mRNA levels of *SOX2* and *CDKN1A* were determined in RA-treated and control spheres by RT-qPCR. Results represent the mean ± SD of three independent experiments; ** *p* < 0.01; **** *p* < 0.0001 compared to controls.

To further elucidate the role of RA in stemness properties, we induced the expansion of putative cancer stem cells (CSCs) forming tumorspheres in SK-ES-1 cells. Cells were cultured in an appropriate medium for the expansion of tumorspheres for 7 days (Figure 4A). To confirm the enrichment of stemness after sphere formation, transcriptional levels of *SOX2* and the neural stem cell marker Nestin (encoded by the *NES* gene) were measured. Tumorspheres exhibited increased SOX2 and Nestin mRNA levels compared to the monolayer cultures (Figure 4B). SOX2 was increased by 3.5-fold (*p* < 0.01), while Nestin was increased 3.1-fold (*p* < 0.0001), thus suggesting an increase in stemness after sphere formation.

RA-induced inhibition caused a reduction in both the number and size of tumorspheres compared to controls (Figure 4C), as the treatment reduced the number of tumorspheres by 21.4% (*p* < 0.01) and sphere size was reduced by 34.1% (*p* < 0.01). To investigate additional molecular effects of RA in the tumorsphere context, the transcriptional levels of *CDKN1A* and *SOX2* were measured in the RA-treated and control spheres. The *CDKN1A* levels increased 2,4-fold (*p* < 0.0001), whereas the *SOX2* levels were not changed by the RA treatment (Figure 4D).

### 3.5. Expression of RA Target Genes That Regulate Cell Cycle Progression and Stemness in ES Tumors

**Figure 5:**
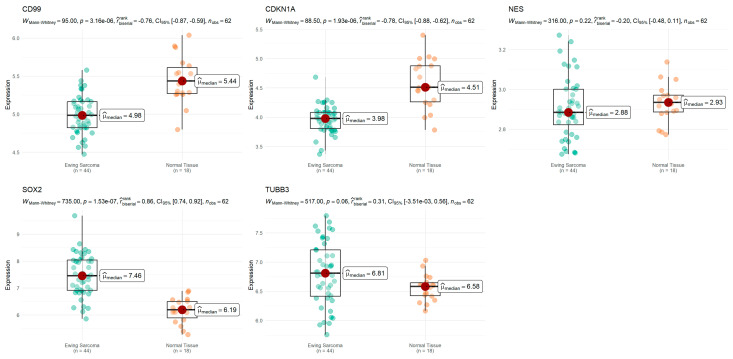
Expression of cell cycle and stemness regulators in ES. Comparisons of gene expression in ES tumors and normal tissue for the genes *CD99*, *CDKN1A*, *NES*, *SOX2*, and *TUBB3* were performed. Expression data from 44 ES tumors and 18 non-tumoral muscle samples from the GSE17674 dataset are shown. Each plot represents a gene, namely *CD99*, *CDKN1A*, *NES*, *SOX2*, and *TUBB3*, where gene expression in tumor and control groups is depicted in boxplots. Statistical analysis for comparisons is shown at the top of the plots.

We compared the expression levels of the *CD99*, *CDKN1A*, *NES*, *SOX2*, and *TUBB3* genes in ES tumor samples and controls consisting of normal muscle tissue. The results are shown in Figure 5. *CD99* and *CDKN1A* showed significantly lower expression levels compared to the controls. In contrast, there were significantly higher levels of *SOX2* in tumors, whereas, for *NES* and *TUBB3*, differences between the groups did not reach statistical significance.

### 3.6. Associations between Gene Expression of Cell Cycle Progression and Stemness Regulators and Prognosis of Patients with ES Tumors

**Figure 6:**
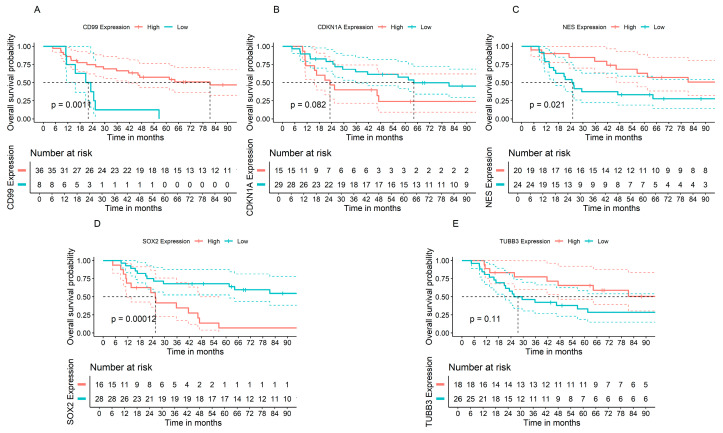
Gene expression of cell cycle and stemness regulators and ES patient survival. OS analysis comparing high versus low expression of the genes (**A**) *CD99*, (**B**) *CDKN1A*, (**C**) *NES*, (**D**) *SOX2*, and (**E**) *TUBB3* were performed. Data show gene expression and OS information from 44 Ewing sarcoma tumors from the GSE17674 dataset; *p* values are embedded in the graphs.

The low expression levels of *CD99* and *NES*, and the high expression levels of *SOX2*, were significantly associated with a worse prognosis assessed by shorter OS. No significant associations of *CDKN1A* and *TUBB3* with OS were found (Figure 6).

## 4. Discussion

RA can regulate stem cell phenotype maintenance versus differentiation in ESCs as well as in CSCs [23,26]. In the present study, we found that RA acutely hinders the viability of SK-ES-1 ES cells; increases expression of *CDKN1A*, *p21*, and *CD99* while reducing expression of *SOX2* and *TUBB3*; and impairs the formation of ES tumorspheres with high expression of *SOX2* and Nestin. It has been previously shown that SK-ES-1 cells express all three types of RARs [27,28]. However, we cannot rule out the possibility that at least some of the RA effects were mediated by receptor-independent mechanisms, such as the activation of transcription factors that do not bind to RAREs [29]. In contrast to what we found in the cell viability and tumorsphere formation assays, the colony-forming ability of SK-ES-1 cells was not significantly affected by RA treatment. A previous study showed that the overexpression of microRNA miR-139-5p significantly reduced invasion but increased the clonogenic capacity of SK-ES-1 cells [30].

Poor differentiation is a feature of ES tumors, which might originate from different types of stem cells. The evidence indicates that ES may arise from either developing neural crest cells or mesenchymal stem cells [20,21,31,32,33,34,35]. ES tumors likely contain a subpopulation of cells with stem cell features. CD133+ cells derived from primary ES tumors are capable of initiating tumors in NOD/SCID mice [36] and display high expression levels of stem cell markers [37,38,39].

The cyclin-dependent kinase (CDK) inhibitor p21 is a well-known tumor suppressor that promotes cell cycle inhibition promoting the G1/S phase, and also acts as a modulator of transcription and apoptosis. In addition to these functions, p21 also regulates differentiation and stemness in CSCs [40], and knockdown of p21 increases the expression of the stemness markers Oct-4 and Nanog in human mesenchymal stem cells [41]. Thus, our observation that RA induced an increase in *CDKN1A* and p21 might be interpreted in the context of stemness regulation, although our experiments were limited to these two markers. Induction of *CDKN1A*/p21 is stimulated by p53, and SK-ES-1 cells harbor a p53 missense mutation which leads to a loss of p53 functioning [42]. However, recent evidence suggests that RA can induce p21 in a p53-independent mechanism in NB4 promyelocytic cells [43], thus a similar process might occur in SK-ES-1 ES cells. Given the well-established role of SOX2 as a stemness marker in CSCs [44,45], the possibility that RA negatively regulates stemness in SK-ES-1 cells is also supported by the reduction we observed in *SOX2* expression in the monolayer, although the same effect was not observed in the tumorspheres. Thus, although RA can impair the formation of SK-ES-1 cell-derived tumorspheres, an in vitro model of the expansion of putative CSCs [24,37,46], we cannot rule out the possibility that the reduction in sphere number involves apoptosis, although we did not examine this process. Consistent with the possibility that tumorspheres were enriched in ES CSCs, increased expression of *SOX2* and *NES*, genes that encode two established CSC markers [38,47,48], was observed in tumorspheres compared to cells cultured in a monolayer.

Other findings seem to contradict our hypothesis that RA would reduce stemness features and promote differentiation in SK-ES-1 cells. In both SH-SY5Y NB [49] and ES [50] cells, neural differentiation with neurite outgrowth is accompanied by increased expression of TUBB3, which is widely used as a marker of neural differentiation. On the other hand, a drug-induced loss of viability in ES cells can be associated with a reduction in TUBB3 expression [51], thus the reduced expression of *TUBB3* we observed might be associated with the antitumor effect of RA in reducing cell viability.

High CD99 expression is a hallmark of ES tumors that contributes to a differential diagnosis, as it is generally considered as a well-established pro-oncogenic factor. Inhibition of CD99 in experimental ES is a strategy that can be used to both impair tumor cell viability and promote neural differentiation [50,52,53,54]. For example, knocking down CD99 in ES cell lines results in neurite outgrowth accompanied by an increased expression of TUBB3 and other markers of neural differentiation [50]. Our finding of increased mRNA expression of CD99 after RA treatment seems to contradict the view that RA can act as a promoter of cell death and differentiation in ES cells. However, in different tumor types, CD99 can act either as a tumor promoter or suppressor, with different isoforms even displaying opposite actions inside the same cell [55]. In addition, CD99 engagement can mediate cell death by either caspase-dependent or -independent mechanisms in ES cells [56,57]. Moreover, in terms of differentiation, high CD99 expression may remain a feature of ES tumors after therapy-induced neural differentiation [58].

Our gene expression analysis indicates a reduction in the transcription of *CD99* and *CDKN1A* and increased levels of *SOX2* in ES tumors, the latter being consistent with the role of stemness in tumor progression. Moreover, we found that a lower expression of *CD99* and *NES* and a higher expression of *SOX2* are associated with a shorter OS in ES patients, suggesting a role for these genes as prognostic markers. It is possible that upregulation of *SOX2* in ES tumors contributes to determining more aggressive disease.

## 5. Conclusions

**Figure 7:**
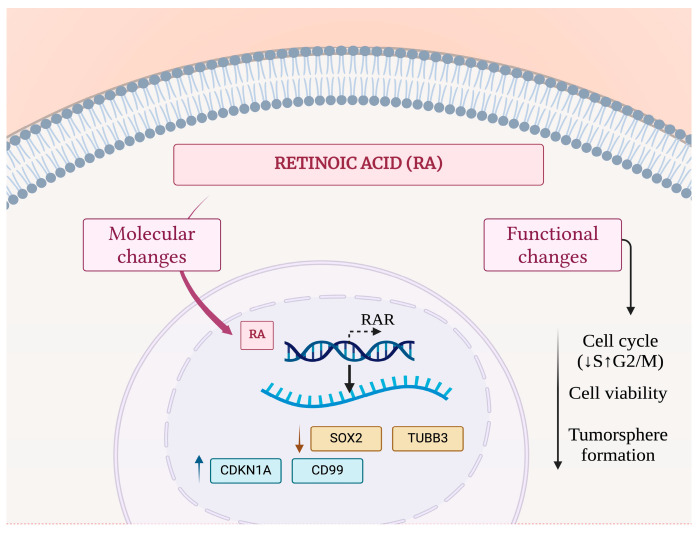
Schematic model depicting molecular and functional actions of RA in ES cells. Exposure to RA leads to a reduction in mRNA or protein levels of SOX2 and TUBB3 and an increase in CDKN1A and CD99. These molecular changes are accompanied by a small reduction of cells in the S phase, G2/M arrest, and reduced cell viability and tumorsphere-forming capability.

In summary, our findings indicate that RA can affect cell function and modulate a limited set of molecular targets associated with stemness features and cell cycle regulation in SK-ES-1 ES cells. These findings are summarized in Figure 7. The expression levels of these genes might influence the prognosis of patients with ES. These early results suggest that RA possibly displays rather complex effects in modulating the maintenance of stem cell characteristics through their actions on multiple molecular targets that are relevant to disease progression in ES.

## Figures and Tables

**Table 1:** Primer sequences used in this study.

Gene	Forward Primer (5′-3′)	Reverse Primer (5′-3′)
*SOX2*	CAGTCTGCAGACCTACATGA	GGGAGGAAGAGGTAACCACAG
*Nestin*	GATCGCTCAGGTCCTGGAAG	GGGGTCCTAGGGAATTGCAG
*TUBB3*	CTCAGGGGCCTTTGGACATC	CAGGCAGTC GCAGTTTTCAC
*CD99*	AAAAGGAGGCAGTGATGGTG	TCCCCTTGTTCTGCATTTTC
*CDKN1A*	ACTCTCAGGGTCGAAAAGGG	CTTCCTGTGGGCGGATTAGG
*ACTB*	AAACTGGAACGG GAAGGTG	AGAGAAGTGGGGTGGCTTTT

## Data Availability

The data that support the findings of this study are available on request from the authors.

## References

[1] Gillespie R.F., Gudas L.J. (2007). Retinoid regulated association of transcriptional co-regulators and the polycomb group protein SUZ12 with the retinoic acid response elements of *Hoxa1*, *RARβ_2_*, and *Cyp26A1* in F9 embryonal carcinoma cells. J. Mol. Biol..

[2] Rhinn M., Dollé P. (2012). Retinoic acid signalling during development. Development.

[3] Chanda B., Ditadi A., Iscove N.N., Kellerm G. (2013). Retinoic acid signaling is essential for embryonic hematopoietic stem cell development. Cell.

[4] Urvalek A.M., Gudas L.J. (2014). Retinoic acid and histone deacetylases regulate epigenetic changes in embryonic stem cells. J. Biol. Chem..

[5] Cuende J., Moreno S., Bolaños J.P., Almeida A. (2008). Retinoic acid downregulates Rae1 leading to APC^Cdh1^ activation and neuroblastoma SH-SY5Y differentiation. Oncogene.

[6] Agholme L., Lindström T., Kågedal K., Marcusson J., Hallbeck M. (2010). An in vitro model for neuroscience: Differentiation of SH-SY5Y cells into cells with morphological and biochemical characteristics of mature neurons. J. Alzheimers Dis..

[7] Korecka J.A., van Kesteren R.E., Blaas E., Spitzer S.O., Kamstra J.H., Smit A.B., Swaab D.F., Verhaagen J., Bossers K. (2013). Phenotypic characterization of retinoic acid differentiated SH-SY5Y cells by transcriptional profiling. PLoS ONE.

[8] Kunzler A., Zeidán-Chuliá F., Gasparotto J., Girardi C.S., Klafke K., Petiz L.L., Bortolin R.C., Rostirolla D.C., Zanotto-Filho A., de Bittencourt Pasquali M.A. (2017). Changes in cell cycle and up-regulation of neuronal markers during SH-SY5Y neurodifferentiation by retinoic acid are mediated by reactive species production and oxidative stress. Mol. Neurobiol..

[9] Thiele C.J., Deutsch L.A., Israel M.A. (1988). The expression of multiple proto-oncogenes is differentially regulated during retinoic acid induced maturation of human neuroblastoma cell lines. Oncogene.

[10] Thiele C.J., Reynolds C.P., Israel M.A. (1985). Decreased expression of N-myc precedes retinoic acid-induced morphological differentiation of human neuroblastoma. Nature.

[11] Villablanca J.G., Khan A.A., Avramis V.I., Seeger R.C., Matthay K.K., Ramsay N.K., Reynolds C.P. (1995). Phase I trial of 13-cis-retinoic acid in children with neuroblastoma following bone marrow transplantation. J. Clin. Oncol..

[12] Matthay K.K., Villablanca J.G., Seeger R.C., Stram D.O., Harris R.E., Ramsay N.K., Swift P., Shimada H., Black C.T., Brodeur G.M. (1999). Treatment of high-risk neuroblastoma with intensive chemotherapy, radiotherapy, autologous bone marrow transplantation, and 13-cis-retinoic acid. Children’s Cancer Group. N. Engl. J. Med..

[13] Adamson P.C., Matthay K.K., O’Brien M., Reaman G.H., Sato J.K., Balis F.M. (2007). A phase 2 trial of all-trans-retinoic acid in combination with interferon-α2a in children with recurrent neuroblastoma or Wilms tumor: A Pediatric Oncology Branch, NCI and Children’s Oncology Group Study. Pediatr. Blood Cancer.

[14] Matthay K.K., Reynolds C.P., Seeger R.C., Shimada H., Adkins E.S., Haas-Kogan D., Gerbing R.B., London W.B., Villablanca J.G. (2009). Long-term results for children with high-risk neuroblastoma treated on a randomized trial of myeloablative therapy followed by 13-cis-retinoic acid: A children’s oncology group study. J. Clin. Oncol..

[15] Jämsä A., Hasslund K., Cowburn R.F., Bäckström A., Vasänge M. (2004). The retinoic acid and brain-derived neurotrophic factor differentiated SH-SY5Y cell line as a model for Alzheimer’s disease-like tau phosphorylation. Biochem. Biophys. Res. Commun..

[16] Constantinescu R., Constantinescu A.T., Reichmann H., Janetzky B. (2007). Neuronal differentiation and long-term culture of the human neuroblastoma line SH-SY5Y. J. Neural Transm. Suppl..

[17] Lopes F.M., Schröder R., da Frota M.L., Zanotto-Filho A., Müller C.B., Pires A.S., Meurer R.T., Colpo G.D., Gelain D.P., Kapczinski F. (2010). Comparison between proliferative and neuron-like SH-SY5Y cells as an in vitro model for Parkinson disease studies. Brain Res..

[18] Lawlor E.R., Thiele C.J. (2012). Epigenetic changes in pediatric solid tumors: Promising new targets. Clin. Cancer Res..

[19] Cavazzana A.O., Miser J.S., Jefferson J., Triche T.J. (1987). Experimental evidence for a neural origin of Ewing’s sarcoma of bone. Am. J. Pathol..

[20] Staege M.S., Hutter C., Neumann I., Foja S., Hattenhorst U.E., Hansen G., Afar D., Burdach S.E. (2004). DNA microarrays reveal relationship of Ewing family tumors to both endothelial and fetal neural crest-derived cells and define novel targets. Cancer Res..

[21] von Levetzow C., Jiang X., Gwye Y., von Levetzow G., Hung L., Cooper A., Hsu J.H., Lawlor E.R. (2011). Modeling initiation of Ewing sarcoma in human neural crest cells. PLoS ONE.

[22] Souza B.K., da Costa Lopez P.L., Menegotto P.R., Vieira I.A., Kersting N., Abujamra A.L., Brunetto A.T., Brunetto A.L., Gregianin L., de Farias C.B. (2018). Targeting histone deacetylase activity to arrest cell growth and promote neural differentiation in Ewing sarcoma. Mol. Neurobiol..

[23] Mezquita B., Mezquita C. (2019). Two opposing faces of retinoic acid: Induction of stemness or induction of differentiation depending on cell-type. Biomolecules.

[24] Wahl J., Bogatyreva L., Boukamp P., Rojewski M., van Valen F., Fiedler J., Hipp N., Debatin K.M., Beltinger C. (2010). Ewing’s sarcoma cells with CD57-associated increase of tumorigenicity and with neural crest-like differentiation capacity. Int. J. Cancer.

[25] Savola S., Klami A., Myllykangas S., Manara C., Scotlandi K., Picci P., Knuutila S., Vakkila J. (2011). High expression of complement component 5 (C5) at tumor site associates with superior survival in Ewing’s sarcoma family of tumour patients. ISRN Oncol..

[26] Brown G. (2023). Retinoic acid receptor regulation of decision-making for cell differentiation. Front. Cell Dev. Biol..

[27] The Human Protein Atlats. https://www.proteinatlas.org/.

[28] Uhlén M., Fagerberg L., Hallström B.M., Lindskog C., Oksvold P., Mardinoglu A., Sivertsson Å., Kampf C., Sjöstedt E., Asplund A. (2015). Proteomics. Tissue-based map of the human proteome. Science.

[29] Kiningham K.K., Silvis A., Claudio P.P.A., Niles R.M. (2018). Receptor independent effects of retinoids. Nutrition and Cancer from Epidemiology to Biology.

[30] Roberto G.M., Delsin L.E.A., Vieira G.M., Silva M.O., Hakime R.G., Gava N.F., Engel E.E., Scrideli C.A., Tone L.G., Brassesco M.S. (2020). ROCK1-PredictedmicroRNAs dysregulation contributes to tumor progression in Ewing sarcoma. Pathol. Oncol. Res..

[31] Riggi N., Cironi L., Provero P., Suvà M.L., Kaloulis K., Garcia-Echeverria C., Hoffmann F., Trumpp A., Stamenkovic I. (2005). Development of Ewing’s sarcoma from primary bone marrow-derived mesenchymal progenitor cells. Cancer Res..

[32] Miyagawa Y., Okita H., Nakaijima H., Horiuchi Y., Sato B., Taguchi T., Toyoda M., Katagiri Y.U., Fujimoto J., Hata J. (2008). Inducible expression of chimeric EWS/ETS proteins confers Ewing’s family tumor-like phenotypes to human mesenchymal progenitor cells. Mol. Cell Biol..

[33] Riggi N., Suvà M.L., Suvà D., Cironi L., Provero P., Tercier S., Joseph J.M., Stehle J.C., Baumer K., Kindler V. (2008). EWS-FLI-1 expression triggers a Ewing’s sarcoma initiation program in primary human mesenchymal stem cells. Cancer Res..

[34] Sole A., Grossetête S., Heintzé M., Babin L., Zaïdi S., Revy P., Renouf B., De Cian A., Giovannangeli C., Pierre-Eugène C. (2021). Unraveling Ewing sarcoma tumorigenesis originating from patient-derived mesenchymal stem cells. Cancer Res..

[35] Hong B., Li Y., Yang R., Dai S., Zhan Y., Zhang W.B., Dong R. (2022). Single-cell transcriptional profiling reveals heterogeneity and developmental trajectories of Ewing sarcoma. J. Cancer Res. Clin. Oncol..

[36] Suvà M.L., Riggi N., Stehle J.C., Baumer K., Tercier S., Joseph J.M., Suvà D., Clément V., Provero P., Cironi L. (2009). Identification of cancer stem cells in Ewing’s sarcoma. Cancer Res..

[37] Awad O., Yustein J.T., Shah P., Gul N., Katuri V., O’Neill A., Kong Y., Brown M.L., Toretsky J.A., Loeb D.M. (2010). High ALDH activity identifies chemotherapy-resistant Ewing’s sarcoma stem cells that retain sensitivity to EWS-FLI1 inhibition. PLoS ONE.

[38] Menendez S.T., Rey V., Martinez-Cruzado L., Gonzalez M.V., Morales-Molina A., Santos L., Blanco V., Alvarez C., Estupiñan O., Allonca E. (2020). SOX2 expression and transcriptional activity identifies a subpopulation of cancer stem cells in sarcoma with prognostic implications. Cancers.

[39] dos Santos R.P., Roesler R., Gregianin L., Brunetto A.T., Jaeger M.C., Brunetto A.L., de Farias C.B. (2023). Cancer stem cells and chemoresistance in Ewing sarcoma. Curr. Stem Cell Res. Ther..

[40] Xiao B.D., Zhao Y.J., Jia X.Y., Wu J., Wang Y.G., Huang F. (2020). Multifaceted p21 in carcinogenesis, stemness of tumor and tumor therapy. World J. Stem Cells.

[41] Yew T.L., Chiu F.Y., Tsai C.C., Chen H.L., Lee W.P., Chen Y.J., Chang M.C., Hung S.C. (2011). Knockdown of p21(Cip1/Waf1) enhances proliferation, the expression of stemness markers, and osteogenic potential in human mesenchymal stem cells. Aging Cell.

[42] Sturm M.J., Henao-Restrepo J.A., Becker S., Proquitté H., Beck J.F., Sonnemann J. (2023). Synergistic anticancer activity of combined ATR and ribonucleotide reductase inhibition in Ewing’s sarcoma cells. J. Cancer Res. Clin. Oncol..

[43] Bocchia M., Xu Q., Wesley U., Xu Y., Korontsvit T., Loganzo F., Albino A.P., Scheinberg D.A. (1997). Modulation of p53, WAF1/p21 and BCL-2 expression during retinoic acid-induced differentiation of NB4 promyelocytic cells. Leuk. Res..

[44] Mamun M.A., Mannoor K., Cao J., Qadri F., Song X. (2020). SOX2 in cancer stemness: Tumor malignancy and therapeutic potentials. J. Mol. Cell Biol..

[45] Schaefer T., Lengerke C. (2020). SOX2 protein biochemistry in stemness, reprogramming, and cancer: The PI3K/AKT/SOX2 axis and beyond. Oncogene.

[46] Cornaz-Buros S., Riggi N., DeVito C., Sarre A., Letovanec I., Provero P., Stamenkovic I. (2014). Targeting cancer stem-like cells as an approach to defeating cellular heterogeneity in Ewing sarcoma. Cancer Res..

[47] Riggi N., Suvà M.L., De Vito C., Provero P., Stehle J.C., Baumer K., Cironi L., Janiszewska M., Petricevic T., Suvà D. (2010). EWS-FLI-1 modulates miRNA145 and SOX2 expression to initiate mesenchymal stem cell reprogramming toward Ewing sarcoma cancer stem cells. Genes Dev..

[48] Neradil J., Veselska R. (2015). Nestin as a marker of cancer stem cells. Cancer Sci..

[49] Xu Y., Kusuyama J., Osana S., Matsuhashi S., Li L., Takada H., Inada H., Nagatomi R. (2023). Lactate promotes neuronal differentiation of SH-SY5Y cells by lactate-responsive gene sets through NDRG3-dependent and -independent manners. J. Biol. Chem..

[50] Rocchi A., Manara M.C., Sciandra M., Zambelli D., Nardi F., Nicoletti G., Garofalo C., Meschini S., Astolfi A., Colombo M.P. (2010). CD99 inhibits neural differentiation of human Ewing sarcoma cells and thereby contributes to oncogenesis. J. Clin. Investig..

[51] Heinen T.E., dos Santos R.P., da Rocha A., dos Santos M.P., Lopez P.L., Silva Filho M.A., Souza B.K., Rivero L.F., Becker R.G., Gregianin L.J. (2016). Trk inhibition reduces cell proliferation and potentiates the effects of chemotherapeutic agents in Ewing sarcoma. Oncotarget.

[52] Guerzoni C., Fiori V., Terracciano M., Manara M.C., Moricoli D., Pasello M., Sciandra M., Nicoletti G., Gellini M., Dominici S. (2015). CD99 triggering in Ewing sarcoma delivers a lethal signal through p53 pathway reactivation and cooperates with doxorubicin. Clin. Cancer Res..

[53] Ventura S., Aryee D.N., Felicetti F., De Feo A., Mancarella C., Manara M.C., Picci P., Colombo M.P., Kovar H., Carè A. (2016). CD99 regulates neural differentiation of Ewing sarcoma cells through miR-34a-Notch-mediated control of NF-κB signaling. Oncogene.

[54] De Feo A., Sciandra M., Ferracin M., Felicetti F., Astolfi A., Pignochino Y., Picci P., Carè A., Scotlandi K. (2019). Exosomes from CD99-deprived Ewing sarcoma cells reverse tumor malignancy by inhibiting cell migration and promoting neural differentiation. Cell Death Dis..

[55] Manara M.C., Pasello M., Scotlandi K. (2018). CD99: A cell surface protein with an oncojanus role in tumors. Genes.

[56] Sohn H.W., Choi E.Y., Kim S.H., Lee I.S., Chung D.H., Sung U.A., Hwang D.H., Cho S.S., Jun B.H., Jang J.J. (1998). Engagement of CD99 induces apoptosis through a calcineurin-independent pathway in Ewing’s sarcoma cells. Am. J. Pathol..

[57] Cerisano V., Aalto Y., Perdichizzi S., Bernard G., Manara M.C., Benini S., Cenacchi G., Preda P., Lattanzi G., Nagy B. (2004). Molecular mechanisms of CD99-induced caspase-independent cell death and cell-cell adhesion in Ewing’s sarcoma cells: Actin and zyxin as key intracellular mediators. Oncogene.

[58] Erdoğan K.E., Deveci M.A., Hakkoymaz Z.R., Gönlüşen G. (2019). Therapy-induced neural differentiation in Ewing’s sarcoma: A case report and review of the literature. Turk. Patoloji. Derg..

